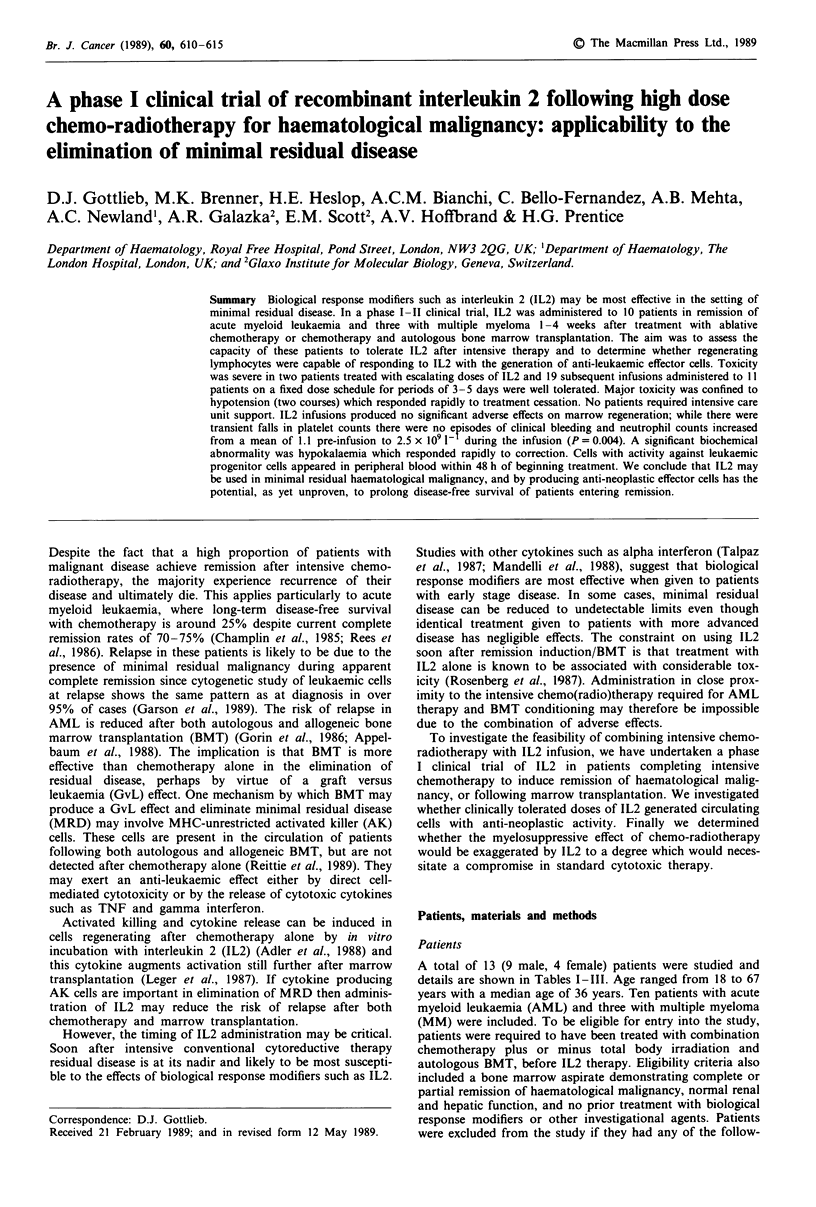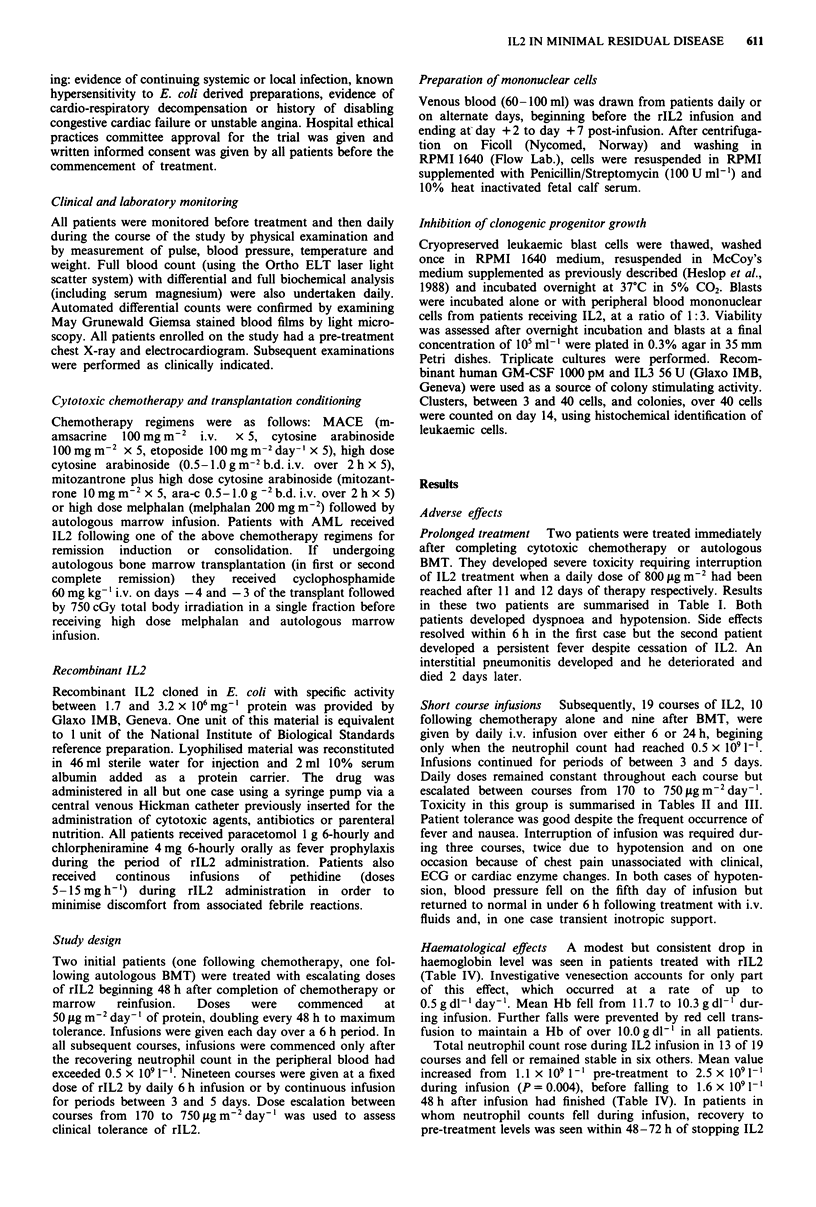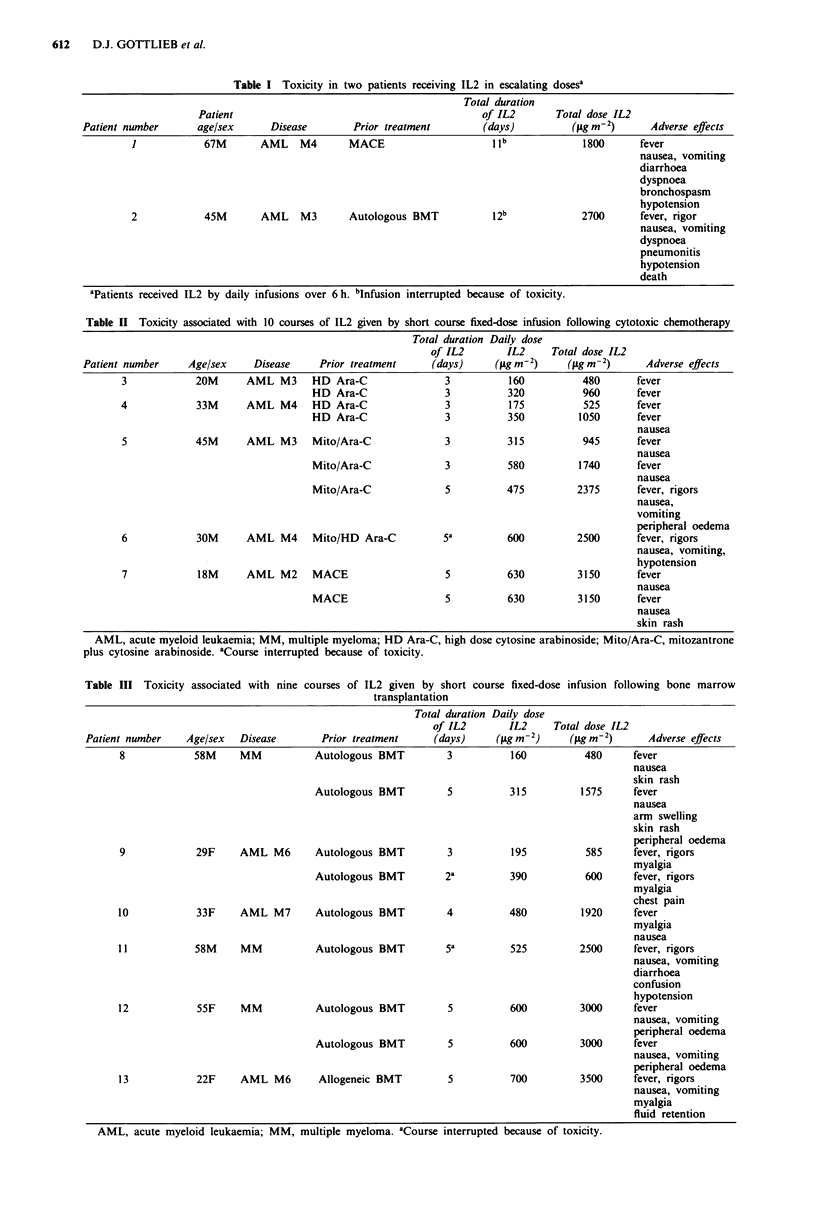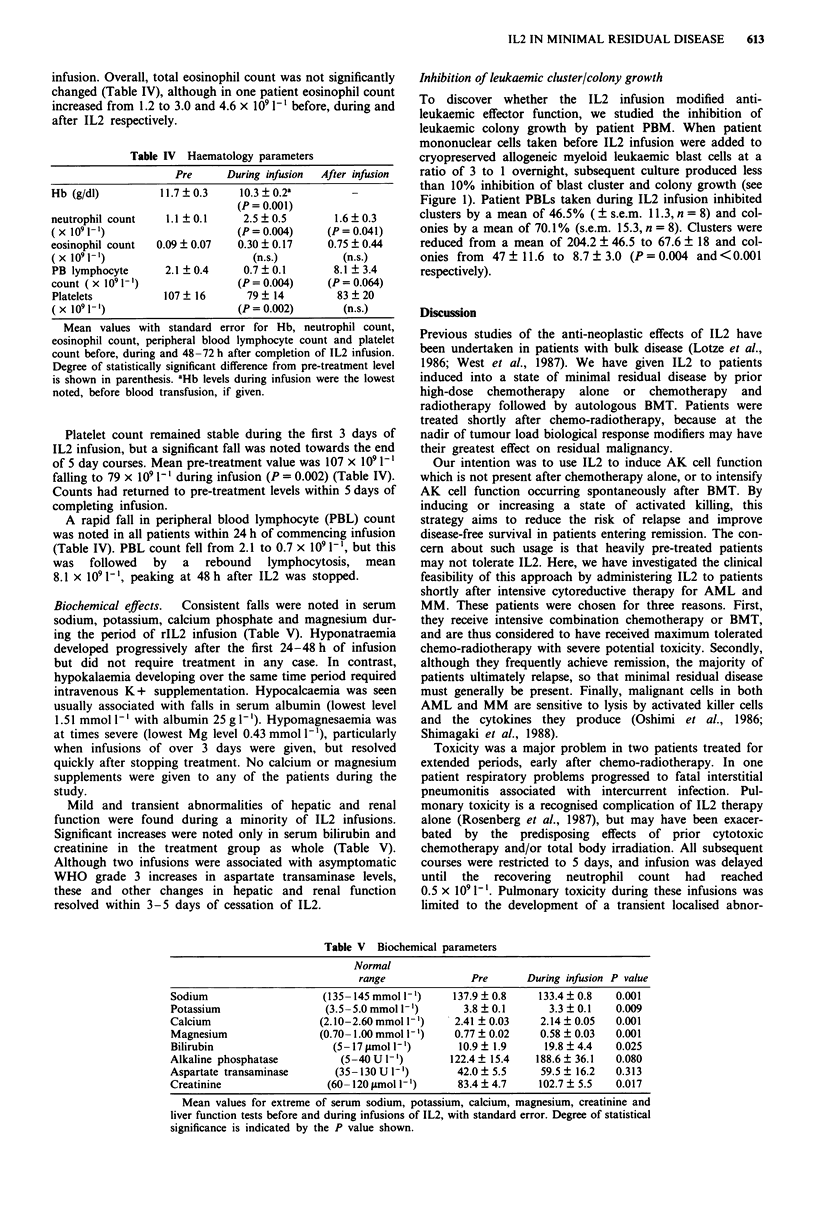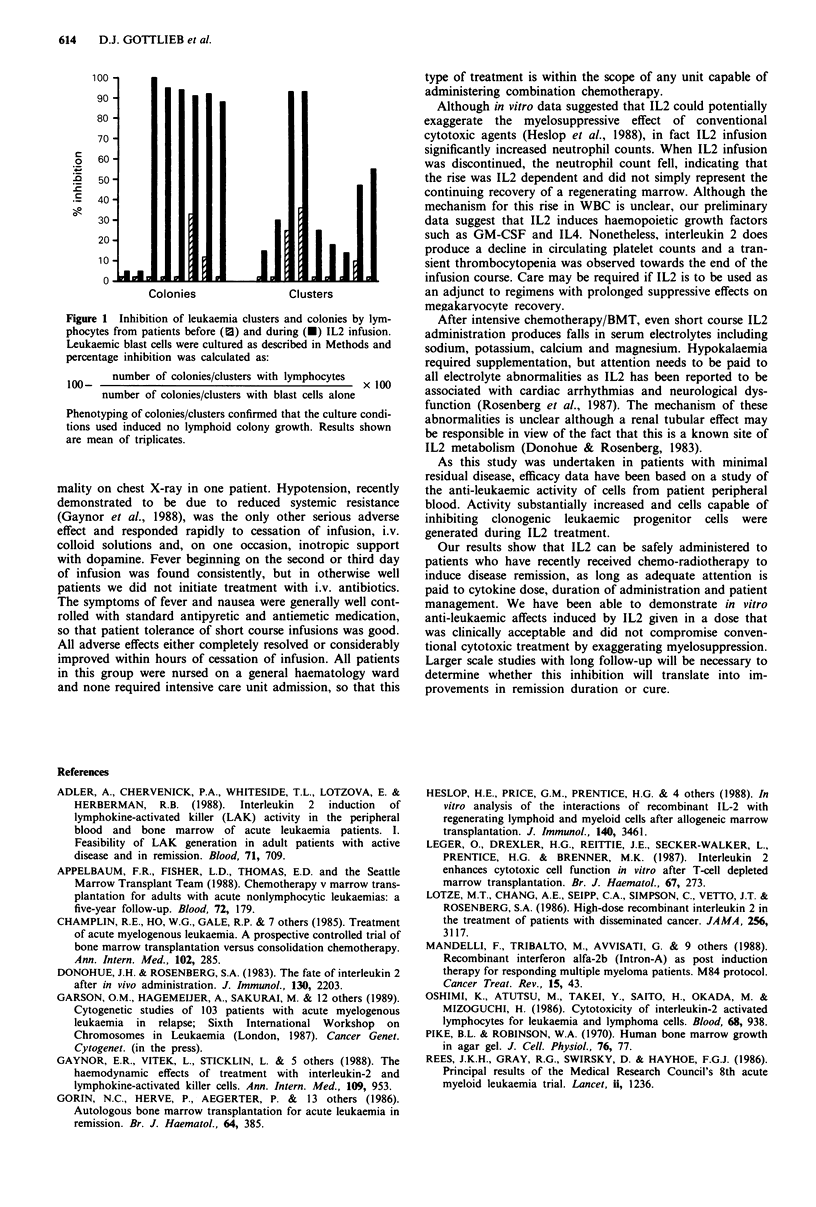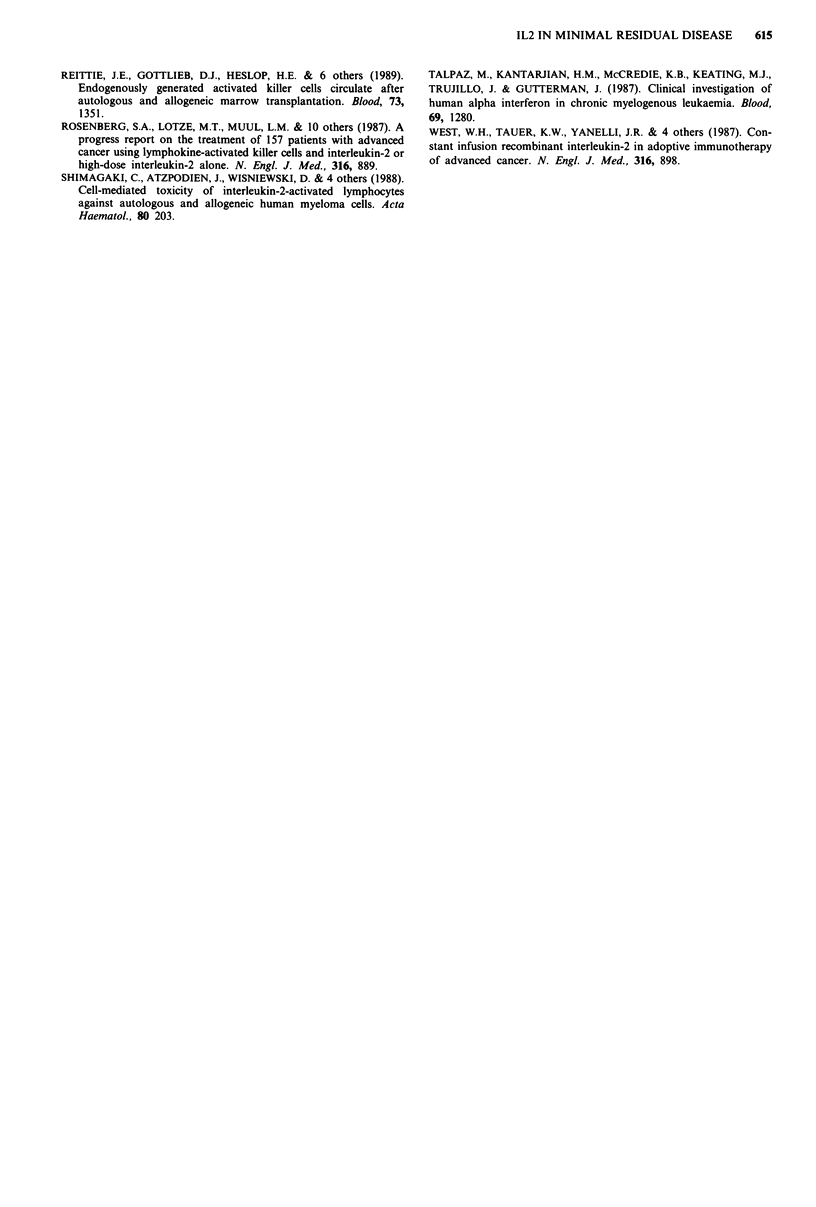# A phase I clinical trial of recombinant interleukin 2 following high dose chemo-radiotherapy for haematological malignancy: applicability to the elimination of minimal residual disease.

**DOI:** 10.1038/bjc.1989.324

**Published:** 1989-10

**Authors:** D. J. Gottlieb, M. K. Brenner, H. E. Heslop, A. C. Bianchi, C. Bello-Fernandez, A. B. Mehta, A. C. Newland, A. R. Galazka, E. M. Scott, A. V. Hoffbrand

**Affiliations:** Department of Haematology, Royal Free Hospital, London, UK.

## Abstract

Biological response modifiers such as interleukin 2 (IL2) may be most effective in the setting of minimal residual disease. In a phase I-II clinical trial, IL2 was administered to 10 patients in remission of acute myeloid leukaemia and three with multiple myeloma 1-4 weeks after treatment with ablative chemotherapy or chemotherapy and autologous bone marrow transplantation. The aim was to assess the capacity of these patients to tolerate IL2 after intensive therapy and to determine whether regenerating lymphocytes were capable of responding to IL2 with the generation of anti-leukaemic effector cells. Toxicity was severe in two patients treated with escalating doses of IL2 and 19 subsequent infusions administered to 11 patients on a fixed dose schedule for periods of 3-5 days were well tolerated. Major toxicity was confined to hypotension (two courses) which responded rapidly to treatment cessation. No patients required intensive care unit support. IL2 infusions produced no significant adverse effects on marrow regeneration; while there were transient falls in platelet counts there were no episodes of clinical bleeding and neutrophil counts increased from a mean of 1.1 pre-infusion to 2.5 x 10(9)l-1 during the infusion (P = 0.004). A significant biochemical abnormality was hypokalaemia which responded rapidly to correction. Cells with activity against leukaemic progenitor cells appeared in peripheral blood within 48 h of beginning treatment. We conclude that IL2 may be used in minimal residual haematological malignancy, and by producing anti-neoplastic effector cells has the potential, as yet unproven, to prolong disease-free survival of patients entering remission.


Br. J. Cancer (1989), 60, 610-615 © The Macmillan Press Ltd., 1989

### A phase I clinical trial of recombinant interleukin 2 following high dose chemo-radiotherapy for haematological malignancy: applicability to the elimination of minimal residual disease

D.J. Gottlieb, M.K. Brenner, H.E. Heslop, A.C.M. Bianchi, C. Bello-Fernandez, A.B. Mehta, A.C. Newland[1], A.R. Galazka[2], E.M. Scott[2], A.V. Hoffbrand & H.G. Prentice

*Department of Haematology, Royal Free Hospital, Pond Street, London, NW3 2QG, UK; [1]Department of Haematology, The London Hospital, London, UK; and [2]Glaxo Institute for Molecular Biology, Geneva, Switzerland.*

**Summary** Biological response modifiers such as interleukin 2 (IL2) may be most effective in the setting of minimal residual disease. In a phase I–II clinical trial, IL2 was administered to 10 patients in remission of acute myeloid leukaemia and three with multiple myeloma 1–4 weeks after treatment with ablative chemotherapy or chemotherapy and autologous bone marrow transplantation. The aim was to assess the capacity of these patients to tolerate IL2 after intensive therapy and to determine whether regenerating lymphocytes were capable of responding to IL2 with the generation of anti-leukaemic effector cells. Toxicity was severe in two patients treated with escalating doses of IL2 and 19 subsequent infusions administered to 11 patients on a fixed dose schedule for periods of 3–5 days were well tolerated. Major toxicity was confined to hypotension (two courses) which responded rapidly to treatment cessation. No patients required intensive care unit support. IL2 infusions produced no significant adverse effects on marrow regeneration; while there were transient falls in platelet counts there were no episodes of clinical bleeding and neutrophil counts increased from a mean of 1.1 pre-infusion to $2.5 \times 10^9 l^{-1}$ during the infusion ($P = 0.004$). A significant biochemical abnormality was hypokalaemia which responded rapidly to correction. Cells with activity against leukaemic progenitor cells appeared in peripheral blood within 48 h of beginning treatment. We conclude that IL2 may be used in minimal residual haematological malignancy, and by producing anti-neoplastic effector cells has the potential, as yet unproven, to prolong disease-free survival of patients entering remission.

Despite the fact that a high proportion of patients with malignant disease achieve remission after intensive chemo-radiotherapy, the majority experience recurrence of their disease and ultimately die. This applies particularly to acute myeloid leukaemia, where long-term disease-free survival with chemotherapy is around 25% despite current complete remission rates of 70–75% (Champlin *et al.*, 1985; Rees *et al.*, 1986). Relapse in these patients is likely to be due to the presence of minimal residual malignancy during apparent complete remission since cytogenetic study of leukaemic cells at relapse shows the same pattern as at diagnosis in over 95% of cases (Garson *et al.*, 1989). The risk of relapse in AML is reduced after both autologous and allogeneic bone marrow transplantation (BMT) (Gorin *et al.*, 1986; Appelbaum *et al.*, 1988). The implication is that BMT is more effective than chemotherapy alone in the elimination of residual disease, perhaps by virtue of a graft versus leukaemia (GvL) effect. One mechanism by which BMT may produce a GvL effect and eliminate minimal residual disease (MRD) may involve MHC-unrestricted activated killer (AK) cells. These cells are present in the circulation of patients following both autologous and allogeneic BMT, but are not detected after chemotherapy alone (Reittie *et al.*, 1989). They may exert an anti-leukaemic effect either by direct cell-mediated cytotoxicity or by the release of cytotoxic cytokines such as TNF and gamma interferon.

Activated killing and cytokine release can be induced in cells regenerating after chemotherapy alone by *in vitro* incubation with interleukin 2 (IL2) (Adler *et al.*, 1988) and this cytokine augments activation still further after marrow transplantation (Leger *et al.*, 1987). If cytokine producing AK cells are important in elimination of MRD then administration of IL2 may reduce the risk of relapse after both chemotherapy and marrow transplantation.

However, the timing of IL2 administration may be critical. Soon after intensive conventional cytoreductive therapy residual disease is at its nadir and likely to be most susceptible to the effects of biological response modifiers such as IL2. Studies with other cytokines such as alpha interferon (Talpaz *et al.*, 1987; Mandelli *et al.*, 1988), suggest that biological response modifiers are most effective when given to patients with early stage disease. In some cases, minimal residual disease can be reduced to undetectable limits even though identical treatment given to patients with more advanced disease has negligible effects. The constraint on using IL2 soon after remission induction/BMT is that treatment with IL2 alone is known to be associated with considerable toxicity (Rosenberg *et al.*, 1987). Administration in close proximity to the intensive chemo(radio)therapy required for AML therapy and BMT conditioning may therefore be impossible due to the combination of adverse effects.

To investigate the feasibility of combining intensive chemo-radiotherapy with IL2 infusion, we have undertaken a phase I clinical trial of IL2 in patients completing intensive chemotherapy to induce remission of haematological malignancy, or following marrow transplantation. We investigated whether clinically tolerated doses of IL2 generated circulating cells with anti-neoplastic activity. Finally we determined whether the myelosuppressive effect of chemo-radiotherapy would be exaggerated by IL2 to a degree which would necessitate a compromise in standard cytotoxic therapy.

### Patients, materials and methods

### *Patients*

A total of 13 (9 male, 4 female) patients were studied and details are shown in Tables I–III. Age ranged from 18 to 67 years with a median age of 36 years. Ten patients with acute myeloid leukaemia (AML) and three with multiple myeloma (MM) were included. To be eligible for entry into the study, patients were required to have been treated with combination chemotherapy plus or minus total body irradiation and autologous BMT, before IL2 therapy. Eligibility criteria also included a bone marrow aspirate demonstrating complete or partial remission of haematological malignancy, normal renal and hepatic function, and no prior treatment with biological response modifiers or other investigational agents. Patients were excluded from the study if they had any of the follow-

Correspondence: D.J. Gottlieb.
Received 21 February 1989; and in revised form 12 May 1989.

ing: evidence of continuing systemic or local infection, known hypersensitivity to *E. coli* derived preparations, evidence of cardio-respiratory decompensation or history of disabling congestive cardiac failure or unstable angina. Hospital ethical practices committee approval for the trial was given and written informed consent was given by all patients before the commencement of treatment.

*Clinical and laboratory monitoring*

All patients were monitored before treatment and then daily during the course of the study by physical examination and by measurement of pulse, blood pressure, temperature and weight. Full blood count (using the Ortho ELT laser light scatter system) with differential and full biochemical analysis (including serum magnesium) were also undertaken daily. Automated differential counts were confirmed by examining May Grunewald Giemsa stained blood films by light microscopy. All patients enrolled on the study had a pre-treatment chest X-ray and electrocardiogram. Subsequent examinations were performed as clinically indicated.

*Cytotoxic chemotherapy and transplantation conditioning*

Chemotherapy regimens were as follows: MACE (m-amsacrine $100 \text{ mg m}^{-2}$ i.v. $\times 5$, cytosine arabinoside $100 \text{ mg m}^{-2} \times 5$, etoposide $100 \text{ mg m}^{-2} \text{ day}^{-1} \times 5$), high dose cytosine arabinoside ($0.5-1.0 \text{ g m}^{-2}$ b.d. i.v. over $2 \text{ h} \times 5$), mitozantrone plus high dose cytosine arabinoside (mitozantrone $10 \text{ mg m}^{-2} \times 5$, ara-c $0.5-1.0 \text{ g}^{-2}$ b.d. i.v. over $2 \text{ h} \times 5$) or high dose melphalan (melphalan $200 \text{ mg m}^{-2}$) followed by autologous marrow infusion. Patients with AML received IL2 following one of the above chemotherapy regimens for remission induction or consolidation. If undergoing autologous bone marrow transplantation (in first or second complete remission) they received cyclophosphamide $60 \text{ mg kg}^{-1}$ i.v. on days $-4$ and $-3$ of the transplant followed by 750 cGy total body irradiation in a single fraction before receiving high dose melphalan and autologous marrow infusion.

*Recombinant IL2*

Recombinant IL2 cloned in *E. coli* with specific activity between 1.7 and $3.2 \times 10^6 \text{ mg}^{-1}$ protein was provided by Glaxo IMB, Geneva. One unit of this material is equivalent to 1 unit of the National Institute of Biological Standards reference preparation. Lyophilised material was reconstituted in 46 ml sterile water for injection and 2 ml 10% serum albumin added as a protein carrier. The drug was administered in all but one case using a syringe pump via a central venous Hickman catheter previously inserted for the administration of cytotoxic agents, antibiotics or parenteral nutrition. All patients received paracetomol 1 g 6-hourly and chlorpheniramine 4 mg 6-hourly orally as fever prophylaxis during the period of rIL2 administration. Patients also received continous infusions of pethidine (doses $5-15 \text{ mg h}^{-1}$) during rIL2 administration in order to minimise discomfort from associated febrile reactions.

*Study design*

Two initial patients (one following chemotherapy, one following autologous BMT) were treated with escalating doses of rIL2 beginning 48 h after completion of chemotherapy or marrow reinfusion. Doses were commenced at $50 \mu\text{g m}^{-2} \text{ day}^{-1}$ of protein, doubling every 48 h to maximum tolerance. Infusions were given each day over a 6 h period. In all subsequent courses, infusions were commenced only after the recovering neutrophil count in the peripheral blood had exceeded $0.5 \times 10^9 \text{ l}^{-1}$. Nineteen courses were given at a fixed dose of rIL2 by daily 6 h infusion or by continuous infusion for periods between 3 and 5 days. Dose escalation between courses from 170 to $750 \mu\text{g m}^{-2} \text{ day}^{-1}$ was used to assess clinical tolerance of rIL2.

*Preparation of mononuclear cells*

Venous blood (60–100 ml) was drawn from patients daily or on alternate days, beginning before the rIL2 infusion and ending at day +2 to day +7 post-infusion. After centrifugation on Ficoll (Nycomed, Norway) and washing in RPMI 1640 (Flow Lab.), cells were resuspended in RPMI supplemented with Penicillin/Streptomycin ($100 \text{ U ml}^{-1}$) and 10% heat inactivated fetal calf serum.

*Inhibition of clonogenic progenitor growth*

Cryopreserved leukaemic blast cells were thawed, washed once in RPMI 1640 medium, resuspended in McCoy's medium supplemented as previously described (Heslop *et al.*, 1988) and incubated overnight at 37°C in 5% $CO_2$. Blasts were incubated alone or with peripheral blood mononuclear cells from patients receiving IL2, at a ratio of 1:3. Viability was assessed after overnight incubation and blasts at a final concentration of $10^5 \text{ ml}^{-1}$ were plated in 0.3% agar in 35 mm Petri dishes. Triplicate cultures were performed. Recombinant human GM-CSF 1000 pM and IL3 56 U (Glaxo IMB, Geneva) were used as a source of colony stimulating activity. Clusters, between 3 and 40 cells, and colonies, over 40 cells were counted on day 14, using histochemical identification of leukaemic cells.

### Results

*Adverse effects*

*Prolonged treatment* Two patients were treated immediately after completing cytotoxic chemotherapy or autologous BMT. They developed severe toxicity requiring interruption of IL2 treatment when a daily dose of $800 \mu\text{g m}^{-2}$ had been reached after 11 and 12 days of therapy respectively. Results in these two patients are summarised in Table I. Both patients developed dyspnoea and hypotension. Side effects resolved within 6 h in the first case but the second patient developed a persistent fever despite cessation of IL2. An interstitial pneumonitis developed and he deteriorated and died 2 days later.

*Short course infusions* Subsequently, 19 courses of IL2, 10 following chemotherapy alone and nine after BMT, were given by daily i.v. infusion over either 6 or 24 h, begining only when the neutrophil count had reached $0.5 \times 10^9 \text{ l}^{-1}$. Infusions continued for periods of between 3 and 5 days. Daily doses remained constant throughout each course but escalated between courses from 170 to $750 \mu\text{g m}^{-2} \text{ day}^{-1}$. Toxicity in this group is summarised in Tables II and III. Patient tolerance was good despite the frequent occurrence of fever and nausea. Interruption of infusion was required during three courses, twice due to hypotension and on one occasion because of chest pain unassociated with clinical, ECG or cardiac enzyme changes. In both cases of hypotension, blood pressure fell on the fifth day of infusion but returned to normal in under 6 h following treatment with i.v. fluids and, in one case transient inotropic support.

*Haematological effects* A modest but consistent drop in haemoglobin level was seen in patients treated with rIL2 (Table IV). Investigative venesection accounts for only part of this effect, which occurred at a rate of up to $0.5 \text{ g dl}^{-1} \text{ day}^{-1}$. Mean Hb fell from 11.7 to $10.3 \text{ g dl}^{-1}$ during infusion. Further falls were prevented by red cell transfusion to maintain a Hb of over $10.0 \text{ g dl}^{-1}$ in all patients.

Total neutrophil count rose during IL2 infusion in 13 of 19 courses and fell or remained stable in six others. Mean value increased from $1.1 \times 10^9 \text{ l}^{-1}$ pre-treatment to $2.5 \times 10^9 \text{ l}^{-1}$ during infusion ($P = 0.004$), before falling to $1.6 \times 10^9 \text{ l}^{-1}$ 48 h after infusion had finished (Table IV). In patients in whom neutrophil counts fell during infusion, recovery to pre-treatment levels was seen within 48–72 h of stopping IL2

**Table I** Toxicity in two patients receiving IL2 in escalating doses[a]

| Patient number | Patient age/sex | Disease | Prior treatment | Total duration of IL2 (days) | Total dose IL2 ($\mu g\ m^{-2}$) | Adverse effects |
|---|---|---|---|---|---|---|
| 1 | 67M | AML M4 | MACE | 11[b] | 1800 | fever<br>nausea, vomiting<br>diarrhoea<br>dyspnoea<br>bronchospasm<br>hypotension |
| 2 | 45M | AML M3 | Autologous BMT | 12[b] | 2700 | fever, rigor<br>nausea, vomiting<br>dyspnoea<br>pneumonitis<br>hypotension<br>death |

[a]Patients received IL2 by daily infusions over 6 h. [b]Infusion interrupted because of toxicity.

**Table II** Toxicity associated with 10 courses of IL2 given by short course fixed-dose infusion following cytotoxic chemotherapy

| Patient number | Age/sex | Disease | Prior treatment | Total duration of IL2 (days) | Daily dose IL2 ($\mu g\ m^{-2}$) | Total dose IL2 ($\mu g\ m^{-2}$) | Adverse effects |
|---|---|---|---|---|---|---|---|
| 3 | 20M | AML M3 | HD Ara-C | 3 | 160 | 480 | fever |
| | | | HD Ara-C | 3 | 320 | 960 | fever |
| 4 | 33M | AML M4 | HD Ara-C | 3 | 175 | 525 | fever |
| | | | HD Ara-C | 3 | 350 | 1050 | fever<br>nausea |
| 5 | 45M | AML M3 | Mito/Ara-C | 3 | 315 | 945 | fever<br>nausea |
| | | | Mito/Ara-C | 3 | 580 | 1740 | fever<br>nausea |
| | | | Mito/Ara-C | 5 | 475 | 2375 | fever, rigors<br>nausea,<br>vomiting<br>peripheral oedema |
| 6 | 30M | AML M4 | Mito/HD Ara-C | 5[a] | 600 | 2500 | fever, rigors<br>nausea, vomiting,<br>hypotension |
| 7 | 18M | AML M2 | MACE | 5 | 630 | 3150 | fever<br>nausea |
| | | | MACE | 5 | 630 | 3150 | fever<br>nausea<br>skin rash |

AML, acute myeloid leukaemia; MM, multiple myeloma; HD Ara-C, high dose cytosine arabinoside; Mito/Ara-C, mitozantrone plus cytosine arabinoside. [a]Course interrupted because of toxicity.

**Table III** Toxicity associated with nine courses of IL2 given by short course fixed-dose infusion following bone marrow transplantation

| Patient number | Age/sex | Disease | Prior treatment | Total duration of IL2 (days) | Daily dose IL2 ($\mu g\ m^{-2}$) | Total dose IL2 ($\mu g\ m^{-2}$) | Adverse effects |
|---|---|---|---|---|---|---|---|
| 8 | 58M | MM | Autologous BMT | 3 | 160 | 480 | fever<br>nausea<br>skin rash |
| | | | Autologous BMT | 5 | 315 | 1575 | fever<br>nausea<br>arm swelling<br>skin rash<br>peripheral oedema |
| 9 | 29F | AML M6 | Autologous BMT | 3 | 195 | 585 | fever, rigors<br>myalgia |
| | | | Autologous BMT | 2[a] | 390 | 600 | fever, rigors<br>myalgia<br>chest pain |
| 10 | 33F | AML M7 | Autologous BMT | 4 | 480 | 1920 | fever<br>myalgia<br>nausea |
| 11 | 58M | MM | Autologous BMT | 5[a] | 525 | 2500 | fever, rigors<br>nausea, vomiting<br>diarrhoea<br>confusion<br>hypotension |
| 12 | 55F | MM | Autologous BMT | 5 | 600 | 3000 | fever<br>nausea, vomiting<br>peripheral oedema |
| | | | Autologous BMT | 5 | 600 | 3000 | fever<br>nausea, vomiting<br>peripheral oedema |
| 13 | 22F | AML M6 | Allogeneic BMT | 5 | 700 | 3500 | fever, rigors<br>nausea, vomiting<br>myalgia<br>fluid retention |

AML, acute myeloid leukaemia; MM, multiple myeloma. [a]Course interrupted because of toxicity.

infusion. Overall, total eosinophil count was not significantly changed (Table IV), although in one patient eosinophil count increased from 1.2 to 3.0 and 4.6 × $10^9 l^{-1}$ before, during and after IL2 respectively.

Table IV Haematology parameters

| | *Pre* | *During infusion* | *After infusion* |
|---|---|---|---|
| Hb (g/dl) | 11.7 ± 0.3 | 10.3 ± 0.2[a] ($P$ = 0.001) | – |
| neutrophil count (× $10^9 l^{-1}$) | 1.1 ± 0.1 | 2.5 ± 0.5 ($P$ = 0.004) | 1.6 ± 0.3 ($P$ = 0.041) |
| eosinophil count (× $10^9 l^{-1}$) | 0.09 ± 0.07 | 0.30 ± 0.17 (n.s.) | 0.75 ± 0.44 (n.s.) |
| PB lymphocyte count (× $10^9 l^{-1}$) | 2.1 ± 0.4 | 0.7 ± 0.1 ($P$ = 0.004) | 8.1 ± 3.4 ($P$ = 0.064) |
| Platelets (× $10^9 l^{-1}$) | 107 ± 16 | 79 ± 14 ($P$ = 0.002) | 83 ± 20 (n.s.) |

Mean values with standard error for Hb, neutrophil count, eosinophil count, peripheral blood lymphocyte count and platelet count before, during and 48–72 h after completion of IL2 infusion. Degree of statistically significant difference from pre-treatment level is shown in parenthesis. [a]Hb levels during infusion were the lowest noted, before blood transfusion, if given.

Platelet count remained stable during the first 3 days of IL2 infusion, but a significant fall was noted towards the end of 5 day courses. Mean pre-treatment value was 107 × $10^9 l^{-1}$ falling to 79 × $10^9 l^{-1}$ during infusion ($P$ = 0.002) (Table IV). Counts had returned to pre-treatment levels within 5 days of completing infusion.

A rapid fall in peripheral blood lymphocyte (PBL) count was noted in all patients within 24 h of commencing infusion (Table IV). PBL count fell from 2.1 to 0.7 × $10^9 l^{-1}$, but this was followed by a rebound lymphocytosis, mean 8.1 × $10^9 l^{-1}$, peaking at 48 h after IL2 was stopped.

*Biochemical effects.* Consistent falls were noted in serum sodium, potassium, calcium phosphate and magnesium during the period of rIL2 infusion (Table V). Hyponatraemia developed progressively after the first 24–48 h of infusion but did not require treatment in any case. In contrast, hypokalaemia developing over the same time period required intravenous K+ supplementation. Hypocalcaemia was seen usually associated with falls in serum albumin (lowest level 1.51 mmol $l^{-1}$ with albumin 25 g $l^{-1}$). Hypomagnesaemia was at times severe (lowest Mg level 0.43 mmol $l^{-1}$), particularly when infusions of over 3 days were given, but resolved quickly after stopping treatment. No calcium or magnesium supplements were given to any of the patients during the study.

Mild and transient abnormalities of hepatic and renal function were found during a minority of IL2 infusions. Significant increases were noted only in serum bilirubin and creatinine in the treatment group as whole (Table V). Although two infusions were associated with asymptomatic WHO grade 3 increases in aspartate transaminase levels, these and other changes in hepatic and renal function resolved within 3–5 days of cessation of IL2.

*Inhibition of leukaemic cluster/colony growth*

To discover whether the IL2 infusion modified anti-leukaemic effector function, we studied the inhibition of leukaemic colony growth by patient PBM. When patient mononuclear cells taken before IL2 infusion were added to cryopreserved allogeneic myeloid leukaemic blast cells at a ratio of 3 to 1 overnight, subsequent culture produced less than 10% inhibition of blast cluster and colony growth (see Figure 1). Patient PBLs taken during IL2 infusion inhibited clusters by a mean of 46.5% (± s.e.m. 11.3, $n$ = 8) and colonies by a mean of 70.1% (s.e.m. 15.3, $n$ = 8). Clusters were reduced from a mean of 204.2 ± 46.5 to 67.6 ± 18 and colonies from 47 ± 11.6 to 8.7 ± 3.0 ($P$ = 0.004 and < 0.001 respectively).

### Discussion

Previous studies of the anti-neoplastic effects of IL2 have been undertaken in patients with bulk disease (Lotze *et al.*, 1986; West *et al.*, 1987). We have given IL2 to patients induced into a state of minimal residual disease by prior high-dose chemotherapy alone or chemotherapy and radiotherapy followed by autologous BMT. Patients were treated shortly after chemo-radiotherapy, because at the nadir of tumour load biological response modifiers may have their greatest effect on residual malignancy.

Our intention was to use IL2 to induce AK cell function which is not present after chemotherapy alone, or to intensify AK cell function occurring spontaneously after BMT. By inducing or increasing a state of activated killing, this strategy aims to reduce the risk of relapse and improve disease-free survival in patients entering remission. The concern about such usage is that heavily pre-treated patients may not tolerate IL2. Here, we have investigated the clinical feasibility of this approach by administering IL2 to patients shortly after intensive cytoreductive therapy for AML and MM. These patients were chosen for three reasons. First, they receive intensive combination chemotherapy or BMT, and are thus considered to have received maximum tolerated chemo-radiotherapy with severe potential toxicity. Secondly, although they frequently achieve remission, the majority of patients ultimately relapse, so that minimal residual disease must generally be present. Finally, malignant cells in both AML and MM are sensitive to lysis by activated killer cells and the cytokines they produce (Oshimi *et al.*, 1986; Shimagaki *et al.*, 1988).

Toxicity was a major problem in two patients treated for extended periods, early after chemo-radiotherapy. In one patient respiratory problems progressed to fatal interstitial pneumonitis associated with intercurrent infection. Pulmonary toxicity is a recognised complication of IL2 therapy alone (Rosenberg *et al.*, 1987), but may have been exacerbated by the predisposing effects of prior cytotoxic chemotherapy and/or total body irradiation. All subsequent courses were restricted to 5 days, and infusion was delayed until the recovering neutrophil count had reached 0.5 × $10^9 l^{-1}$. Pulmonary toxicity during these infusions was limited to the development of a transient localised abnor-

Table V Biochemical parameters

| | *Normal range* | *Pre* | *During infusion* | *P value* |
|---|---|---|---|---|
| Sodium | (135–145 mmol $l^{-1}$) | 137.9 ± 0.8 | 133.4 ± 0.8 | 0.001 |
| Potassium | (3.5–5.0 mmol $l^{-1}$) | 3.8 ± 0.1 | 3.3 ± 0.1 | 0.009 |
| Calcium | (2.10–2.60 mmol $l^{-1}$) | 2.41 ± 0.03 | 2.14 ± 0.05 | 0.001 |
| Magnesium | (0.70–1.00 mmol $l^{-1}$) | 0.77 ± 0.02 | 0.58 ± 0.03 | 0.001 |
| Bilirubin | (5–17 μmol $l^{-1}$) | 10.9 ± 1.9 | 19.8 ± 4.4 | 0.025 |
| Alkaline phosphatase | (5–40 U $l^{-1}$) | 122.4 ± 15.4 | 188.6 ± 36.1 | 0.080 |
| Aspartate transaminase | (35–130 U $l^{-1}$) | 42.0 ± 5.5 | 59.5 ± 16.2 | 0.313 |
| Creatinine | (60–120 μmol $l^{-1}$) | 83.4 ± 4.7 | 102.7 ± 5.5 | 0.017 |

Mean values for extreme of serum sodium, potassium, calcium, magnesium, creatinine and liver function tests before and during infusions of IL2, with standard error. Degree of statistical significance is indicated by the $P$ value shown.

**Figure 1** Inhibition of leukaemia clusters and colonies by lymphocytes from patients before (▨) and during (■) IL2 infusion. Leukaemic blast cells were cultured as described in Methods and percentage inhibition was calculated as:

$$100 - \frac{\text{number of colonies/clusters with lymphocytes}}{\text{number of colonies/clusters with blast cells alone}} \times 100$$

Phenotyping of colonies/clusters confirmed that the culture conditions used induced no lymphoid colony growth. Results shown are mean of triplicates.

mality on chest X-ray in one patient. Hypotension, recently demonstrated to be due to reduced systemic resistance (Gaynor *et al.*, 1988), was the only other serious adverse effect and responded rapidly to cessation of infusion, i.v. colloid solutions and, on one occasion, inotropic support with dopamine. Fever beginning on the second or third day of infusion was found consistently, but in otherwise well patients we did not initiate treatment with i.v. antibiotics. The symptoms of fever and nausea were generally well controlled with standard antipyretic and antiemetic medication, so that patient tolerance of short course infusions was good. All adverse effects either completely resolved or considerably improved within hours of cessation of infusion. All patients in this group were nursed on a general haematology ward and none required intensive care unit admission, so that this type of treatment is within the scope of any unit capable of administering combination chemotherapy.

Although *in vitro* data suggested that IL2 could potentially exaggerate the myelosuppressive effect of conventional cytotoxic agents (Heslop *et al.*, 1988), in fact IL2 infusion significantly increased neutrophil counts. When IL2 infusion was discontinued, the neutrophil count fell, indicating that the rise was IL2 dependent and did not simply represent the continuing recovery of a regenerating marrow. Although the mechanism for this rise in WBC is unclear, our preliminary data suggest that IL2 induces haemopoietic growth factors such as GM-CSF and IL4. Nonetheless, interleukin 2 does produce a decline in circulating platelet counts and a transient thrombocytopenia was observed towards the end of the infusion course. Care may be required if IL2 is to be used as an adjunct to regimens with prolonged suppressive effects on megakaryocyte recovery.

After intensive chemotherapy/BMT, even short course IL2 administration produces falls in serum electrolytes including sodium, potassium, calcium and magnesium. Hypokalaemia required supplementation, but attention needs to be paid to all electrolyte abnormalities as IL2 has been reported to be associated with cardiac arrhythmias and neurological dysfunction (Rosenberg *et al.*, 1987). The mechanism of these abnormalities is unclear although a renal tubular effect may be responsible in view of the fact that this is a known site of IL2 metabolism (Donohue & Rosenberg, 1983).

As this study was undertaken in patients with minimal residual disease, efficacy data have been based on a study of the anti-leukaemic activity of cells from patient peripheral blood. Activity substantially increased and cells capable of inhibiting clonogenic leukaemic progenitor cells were generated during IL2 treatment.

Our results show that IL2 can be safely administered to patients who have recently received chemo-radiotherapy to induce disease remission, as long as adequate attention is paid to cytokine dose, duration of administration and patient management. We have been able to demonstrate *in vitro* anti-leukaemic affects induced by IL2 given in a dose that was clinically acceptable and did not compromise conventional cytotoxic treatment by exaggerating myelosuppression. Larger scale studies with long follow-up will be necessary to determine whether this inhibition will translate into improvements in remission duration or cure.

### References

ADLER, A., CHERVENICK, P.A., WHITESIDE, T.L., LOTZOVA, E. & HERBERMAN, R.B. (1988). Interleukin 2 induction of lymphokine-activated killer (LAK) activity in the peripheral blood and bone marrow of acute leukaemia patients. I. Feasibility of LAK generation in adult patients with active disease and in remission. *Blood*, **71**, 709.

APPELBAUM, F.R., FISHER, L.D., THOMAS, E.D. and the Seattle Marrow Transplant Team (1988). Chemotherapy v marrow transplantation for adults with acute nonlymphocytic leukaemias: a five-year follow-up. *Blood*, **72**, 179.

CHAMPLIN, R.E., HO, W.G., GALE, R.P. & 7 others (1985). Treatment of acute myelogenous leukaemia. A prospective controlled trial of bone marrow transplantation versus consolidation chemotherapy. *Ann. Intern. Med.*, **102**, 285.

DONOHUE, J.H. & ROSENBERG, S.A. (1983). The fate of interleukin 2 after *in vivo* administration. *J. Immunol.*, **130**, 2203.

GARSON, O.M., HAGEMEIJER, A., SAKURAI, M. & 12 others (1989). Cytogenetic studies of 103 patients with acute myelogenous leukaemia in relapse; Sixth International Workshop on Chromosomes in Leukaemia (London, 1987). *Cancer Genet. Cytogenet.* (in the press).

GAYNOR, E.R., VITEK, L., STICKLIN, L. & 5 others (1988). The haemodynamic effects of treatment with interleukin-2 and lymphokine-activated killer cells. *Ann. Intern. Med.*, **109**, 953.

GORIN, N.C., HERVE, P., AEGERTER, P. & 13 others (1986). Autologous bone marrow transplantation for acute leukaemia in remission. *Br. J. Haematol.*, **64**, 385.

HESLOP, H.E., PRICE, G.M., PRENTICE, H.G. & 4 others (1988). *In vitro* analysis of the interactions of recombinant IL-2 with regenerating lymphoid and myeloid cells after allogeneic marrow transplantation. *J. Immunol.*, **140**, 3461.

LEGER, O., DREXLER, H.G., REITTIE, J.E., SECKER-WALKER, L., PRENTICE, H.G. & BRENNER, M.K. (1987). Interleukin 2 enhances cytotoxic cell function *in vitro* after T-cell depleted marrow transplantation. *Br. J. Haematol.*, **67**, 273.

LOTZE, M.T., CHANG, A.E., SEIPP, C.A., SIMPSON, C., VETTO, J.T. & ROSENBERG, S.A. (1986). High-dose recombinant interleukin 2 in the treatment of patients with disseminated cancer. *JAMA*, **256**, 3117.

MANDELLI, F., TRIBALTO, M., AVVISATI, G. & 9 others (1988). Recombinant interferon alfa-2b (Intron-A) as post induction therapy for responding multiple myeloma patients. M84 protocol. *Cancer Treat. Rev.*, **15**, 43.

OSHIMI, K., ATUTSU, M., TAKEI, Y., SAITO, H., OKADA, M. & MIZOGUCHI, H. (1986). Cytotoxicity of interleukin-2 activated lymphocytes for leukaemia and lymphoma cells. *Blood*, **68**, 938.

PIKE, B.L. & ROBINSON, W.A. (1970). Human bone marrow growth in agar gel. *J. Cell. Physiol.*, **76**, 77.

REES, J.K.H., GRAY, R.G., SWIRSKY, D. & HAYHOE, F.G.J. (1986). Principal results of the Medical Research Council's 8th acute myeloid leukaemia trial. *Lancet*, **ii**, 1236.

REITTIE, J.E., GOTTLIEB, D.J., HESLOP, H.E. & 6 others (1989). Endogenously generated activated killer cells circulate after autologous and allogeneic marrow transplantation. *Blood*, **73**, 1351.

ROSENBERG, S.A., LOTZE, M.T., MUUL, L.M. & 10 others (1987). A progress report on the treatment of 157 patients with advanced cancer using lymphokine-activated killer cells and interleukin-2 or high-dose interleukin-2 alone. *N. Engl. J. Med.*, **316**, 889.

SHIMAGAKI, C., ATZPODIEN, J., WISNIEWSKI, D. & 4 others (1988). Cell-mediated toxicity of interleukin-2-activated lymphocytes against autologous and allogeneic human myeloma cells. *Acta Haematol.*, **80** 203.

TALPAZ, M., KANTARJIAN, H.M., MCCREDIE, K.B., KEATING, M.J., TRUJILLO, J. & GUTTERMAN, J. (1987). Clinical investigation of human alpha interferon in chronic myelogenous leukaemia. *Blood*, **69**, 1280.

WEST, W.H., TAUER, K.W., YANELLI, J.R. & 4 others (1987). Constant infusion recombinant interleukin-2 in adoptive immunotherapy of advanced cancer. *N. Engl. J. Med.*, **316**, 898.